# Machine learning, statistical learning and the future of biological research in psychiatry

**DOI:** 10.1017/S0033291716001367

**Published:** 2016-07-13

**Authors:** R. Iniesta, D. Stahl, P. McGuffin

**Affiliations:** 1 Social, Genetic and Developmental Psychiatry Centre, Institute of Psychiatry, Psychology and Neuroscience, King's College London, UK; 2 Department of Biostatistics, Institute of Psychiatry, Psychology and Neuroscience, King's College London, UK

**Keywords:** Machine learning, outcome prediction, personalized medicine, predictive modelling, statistical learning

## Abstract

Psychiatric research has entered the age of ‘Big Data’. Datasets now routinely involve thousands of heterogeneous variables, including clinical, neuroimaging, genomic, proteomic, transcriptomic and other ‘omic’ measures. The analysis of these datasets is challenging, especially when the number of measurements exceeds the number of individuals, and may be further complicated by missing data for some subjects and variables that are highly correlated. Statistical learning-based models are a natural extension of classical statistical approaches but provide more effective methods to analyse very large datasets. In addition, the predictive capability of such models promises to be useful in developing decision support systems. That is, methods that can be introduced to clinical settings and guide, for example, diagnosis classification or personalized treatment. In this review, we aim to outline the potential benefits of statistical learning methods in clinical research. We first introduce the concept of Big Data in different environments. We then describe how modern statistical learning models can be used in practice on Big Datasets to extract relevant information. Finally, we discuss the strengths of using statistical learning in psychiatric studies, from both research and practical clinical points of view.

## The ‘data explosion’ in psychiatry

Once the problem of psychiatric research was that there were not enough data. Now, with the pace of technological advances that have occurred in the present century in neuroimaging, genomics, transcriptomics, proteomics and all the other ‘omics’, we are in danger of being overwhelmed by a volume of data that the human brain, aided only by ‘traditional’ statistical methods, cannot assimilate and integrate. For example, genome-wide association studies (GWAS) now typically and routinely generate millions of data points on tens of thousands of subjects. This has led to some breath-taking advances, notably the finding, based on data from 37 000 patients, that over 100 different genetic loci have a role in schizophrenia (Schizophrenia Working Group of the Psychiatric Genomics, 2014). Similar large-scale studies are underway for other common disorders and, in the UK alone, plans are in place to sequence the entire genomes of 100 000 subjects (http://www.genomicsengland.co.uk). The standard statistical analyses of GWAS are, in principle, straightforward involving χ^2^ tests comparing genetic marker frequencies in cases and controls and applying a stringent correction for multiple testing. However GWAS findings tend to throw up many other problems that will not be solved by such simple analyses. For example, none of the hundred-plus genome-wide significant loci is necessary or sufficient to cause schizophrenia, so this poses a series of new questions. What combinations of loci in interplay with what environmental insults might be useful in predicting who becomes affected in at-risk groups? What combinations of loci relate to what symptom patterns, courses’ outcomes or responses to treatment? What combinations of genetic loci influence structural or functional brain-imaging characteristics? (This is a particularly thorny problem since imaging studies typically generate many more data points even than genomics.) We suggest that a set of solutions to 21st century psychiatry's information overload problems is offered by machine learning (ML) and in particular from a branch that is now often called statistical learning (SL).

## A world of Big Datasets and the role of SL

Although many of us are probably unaware of it, SL is happening all around us. Social media developers, committed to retaining their users and encouraging their online activity are constantly storing information about users and their daily actions in huge datasets, and employ specific methods of analysis designed to deduce what users might ‘like’ next (e.g. new people to incorporate as ‘friends’ or pages that might be of interest). In a similar way, commercial websites such as Amazon aim to predict what product we would next like to buy by thoroughly collecting our history of shopping baskets in databases and investigating our pattern of shopping and comparing it with persons of similar shopping patterns. Another and more individual example is that many of us now use voice recognition software as an alternative to manual typing. Such software not only learns to interpret what we say into printed word but also learns our personal vocabulary, idioms and patterns of expression.

The datasets involved in such processes have three main aspects in common: they occupy vast amounts of computer memory, measured in Terabytes (trillions of bytes), they are heterogeneous containing information coming from a variety of sources, for example a combination of text messages, images and videos, and they are constantly and quickly being updated with new information. These three aspects have been proposed by some authors as the main characteristics of Big Datasets and summarized as the three Vs – volume, variety and velocity (Laney, 2001).

Large-scale datasets from clinical trials and cohort studies, electronic health records or national health registries are becoming increasingly available in biomedical research. They are becoming the focus of research studies that aim to better understand genotype–phenotype relationships, find factors that can predict disease risk, discover profiles of patients that are better responders to a treatment and discover or define disease categories. In general, these datasets meet the three Vs definition, so we can state that biomedical research has definitely entered the Big Data world.

The urgent need of methods that can help to understand such complex Big Datasets has led to a revolution in statistical sciences. Whereas statistics has focused primarily on what conclusions can be inferred from data, Big Datasets have raised other questions about what computational architectures and algorithms can be more efficient to extract maximum information from data in a computationally tractable way (Mitchell, 2006). ML (Soler Artigas *et al.* 2011) refers to a discipline that offers a set of tools built within the intersection of computer sciences and statistics that are capable of coping with the requirements of the Big Data world. These ‘statistical-computational’ systems improve their performance at particular tasks by experience (Mitchell, 1997, 2006; Soler Artigas *et al*. 2011), which is they are capable of learning from data.

Other terms commonly used in the area of ML, but showing slight conceptual differences include artificial intelligence, which encompasses natural language processing, knowledge representation and automated reasoning (Barr *et al.* 1981; Ripley, 1996; Russell & Norvig, 2010), deep learning, a new type of ML algorithm based on neural networks with the aim of discerning higher level features from data (LeCun *et al.* 2015). Other approaches include pattern recognition, a branch of ML focused on the recognition of patterns and regularities in data (Bishop, 2006) and data mining, the process of exploring data in search of consistent patterns and/or systematic relationships between variables (Hand *et al.* 2001).

SL is a fairly recently coined term (Hastie *et al.* 2009) that refers to a vast set of statistical and computational methods to understand complex data. These are based on a range of approaches, from classical concepts belonging to the first half of the 20th century such as linear regression modelling and discriminant analysis, to the latest advanced computational-based approaches including modern ML. Hence SL is a broad term that emphasizes the essential role of statistics within ML in the context of Big Data analysis.

## Learning from data

The methods that underlie SL *learn from data*, i.e. they are able to explore and retain significant structure from data that is replicable across different samples extracted from the same population. Broadly there are three categories of learning from data. The first concerns ‘supervised’ learning (Hastie *et al.* 2009), which typically involves building an algorithm that uses as input a dataset of candidate predictors known as *features* or *attributes* (e.g. age, cancer staging, hospital admissions) and is able to estimate a specific *outcome* (e.g. 6-month survival for cancer patients). Supervised learning includes classification and regression problems. In a classification problem the aim is to determine what category something belongs to, after seeing a number of examples of things from the relevant categories.

The second major category concerns ‘unsupervised’ learning (Ghahramani, 2003) when there is no predefined outcome to be predicted. The task here is deriving an algorithm able to explore data patterns and to discover structure, for example groups of patients who share similar clinical or test result profiles. The two cornerstones of unsupervised learning are clustering (Everitt *et al.* 2010), and dimensionality reduction (Lu *et al.* 2013) which includes principal components analysis and factor analysis. These methods have found important applications in medical research, particularly in psychiatric studies (Ochoa *et al.* 2013; Brodersen *et al.* 2014).

A third category, known as ‘semisupervised’ learning (Zhu & Goldberg, 2009) combines insights from supervised and unsupervised methods by exploring observations where the outcome (or label) is known only for a small amount of data (e.g. the study of the profile of patients that response positively or negatively to a drug, combined with the study of patients with unknown treatment outcome).

In the remainder of this review we will focus on supervised learning problems. Here the outcome is a variable taking either a number of levels that are often called ‘classes’ or ‘labels’ (e.g. relapsing or non-relapsing of a condition), or a quantitative value (e.g. response to treatment as measured by a rating scale). Thus when we talk about ‘labelled data’ we refer to a set of observations for which the outcome is known.

**Fig. 1.**
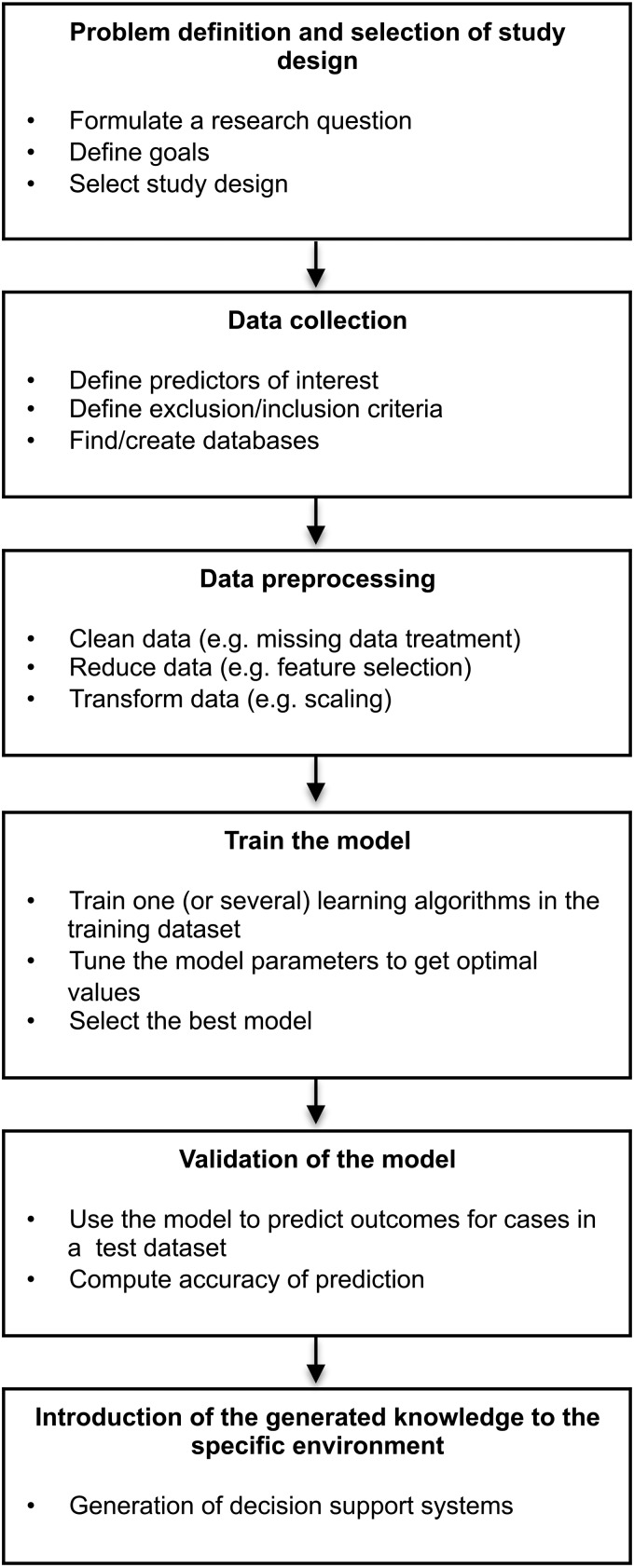
Main steps of the learning process.

The main stages of the learning process are given below (see Fig. 1).

### Definition of the problem and selection of study design

The problem we aim to solve needs to be precisely defined and well understood. As with all research the starting point is critical review of the previous knowledge in the area, formulation of a research question and choice of appropriate study design (Katz, 2006). For example, a longitudinal collection of patients’ data may allow investigation of the risk of an occurrence or relapse concerning a disease over time. Designs such as the case-control that collect data of disease and healthy individuals at just one point in time, will be appropriate to test the ability of a set of factors in predicting a diagnosis.

### Data collection and pre-processing

Ideally, quality data will include a well-defined selection of patients, and a rigorous collection of relevant predictors and outcomes. Before analysis, the main steps of data pre-processing include data cleaning, data reduction and data transformation.

*Cleaning* refers to the treatment of missing data, a common problem in psychiatric research, and this is important as inadequate missing data treatment may lead to an overestimation of prediction accuracy (Batista & Monard, 2002). Discarding individuals or variables with missing values (‘the complete-case analysis’) may bias analysis if the units with missing values differ systematically from the completely observed cases, especially if percentage of missingness is high. A preferable approach may be to estimate or ‘impute’ missing values using either classical statistics or SL. SL methods (e.g. tree-based methods; Ding *et al.* 2010) (Table 1) are free of assumptions and have been found to outperform classical statistical methods of imputation. For example, the methods based on SL techniques were the most suited for the imputation of missing values in a study aiming to predict cancer recurrence, and led to a significant enhancement of prognosis accuracy compared to imputation methods based on statistical procedures (Jerez *et al.* 2010).

**Table 1.** Main properties of a set of selected statistical learning algorithms

Machine learning algorithm	Details
General linear regression models (GLM)	• A very simple approach based on specifying a linear combination of predictors to predict a dependent variable (Hastie *et al.* 2009) • Coefficients of the model are a measure of the strength of effect of each predictor on the outcome • Include linear and logistic regression models (Hosmer *et al.* 2013) • Can present overfitting and multicollinearity in high-dimensional problems
Elastic net models	• Extension of general linear regression models (Zou, 2005) • Explore a large number of predictors to keep the best set of variables in predicting the outcome. This is an internal feature selection method that avoids too complex models and thus prevents of overfitting • Strongly correlated predictors are selected (or discarded) together (what is known as a ‘grouping effect’). This is especially interesting in an exploratory research where the full list of predictors to explore can result equally relevant and meaningful • Coefficients can be interpreted as in a general linear model • Lasso and ridge regression are particular cases (Tibshirani, 1994)
Naive Bayes	• Family of simple classifiers based on applying the Bayes’ Theorem (Russell & Norvig, 2010) (see Fig. 2) • Assumes (*a*) the value of a particular feature is independent of the value of any other feature and (*b*) a probability density for numeric predictors • Gives the probability of taking a specific outcome value for unseen cases
Classification and Regression Trees (CART)	• A tree is a flowchart like structure (Breiman, 1984), built by repeatedly splitting data into subsets based on a feature value test (see Fig. 2). Each terminal node (‘leaf’) holds a label • Allows modeling complex nonlinear relationships • Relatively fast to construct and produce interpretable models • Performs internal feature selection as an integral part of the procedure
Random forest	• Offers a rule to combine individual decision trees (Breiman, 2001*b*) • Multiple tree models are built using different randomly selected subsamples of the full dataset and different initial variables. Then they are aggregated and the most popular outcome value is voted • Good to control overfitting and improve stability and inaccuracy
Support vector machines (SVM)	• Classifier method that constructs hyperplanes in a multidimensional space that separates cases of different outcome values (Cortes & Vapnik, 1995; Scholkopf *et al.* 2003) (see Fig. 2) • A new case is classified depending on his relative position to the decision boundary • Allows modeling complex non-linear relationships. A set of transformations called ‘kernels’ is used to map data and make them linearly separable • Understanding the contribution of each predictor to outcome prediction is not straightforward and must be explored using specific methods (Altmann *et al.* 2010)
Artificial neural networks (ANN)	• A computer system that simulates the essential features of neurons and their interconnections with the aim of processing information the same way as real networks of neurons do (Ripley, 1996) (see Fig. 2) • A neuron receives inputs from other neurons through dendrites, processes them, and delivers an outcome through axon. Connections between neurons are weighted during training. Input nodes are features, output nodes are outcomes. Between them there are hidden layers that are formed of a set of nodes • Allow modelling complex nonlinear relationships • Less likely to be used in medical research due to the lack of interpretability of (*a*) the equations that ANNs generate and (*b*) the transformation of the original dataset into numerical values that ANNs apply

All methods listed above can be used for classification (categorical outcome) and for regression (quantitative outcomes) problems. All of them can handle multiple continuous and categorical predictors.

*Data reduction* involves obtaining a reduced representation of the data volume that can achieve the same (or almost the same) analytical results. By creating new features as a result of the aggregation or eliminating features that are not meaningful for prediction (‘feature selection’) tasks can be made more computationally tractable. Reducing the number of features also makes models more easily interpretable. This point is critical for the success of a predictive algorithm, especially if there are thousands of features at the outset (Guyon, 2003; Witten & Tibshirani, 2010). Feature reduction can be performed as a part of pre-processing or during the modelling step using algorithms that perform an internal feature selection (elastic net regression; Zou & Hastie, 2005) or Classification and Regression Tree (CART) algorithms (Rokach & Maimon, 2008) (Table 1). The latter will usually improve reliability and increase confidence in selected features (Caruana & Niculescu-Mizil, 2006; Krstajic *et al.* 2014).

**Fig. 2.**
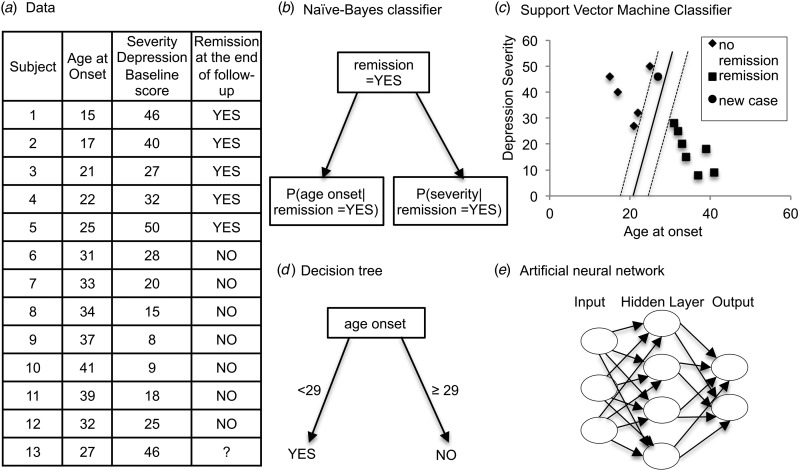
(*a*) Data simulated from a follow-up study of major depression patients. Age of depression onset (years) and the MADRS score at baseline ranging from 0 to 60 (0–6, normal; 7–19, mild depression; 20–34, moderate depression; >34, severe depression) are the predictor variables. The outcome is remission status at the end of the follow-up (YES or NO). (*b*) The Naive Bayes classifier is often represented as this type of graph. The direction of the arrows states that each class causes certain features, with a certain probability. (*c*) A hyper plane (a line, in dimension 2) is built at a maximal distance to every dashed line (called margin). A new case (point) will be classified as remission or non-remission depending on his relative position to the line (aka decision boundary). (*d*) A simple decision tree suggesting that patients with age of onset lower than 29 are more likely to reach a remission. (*e*) Each node represents an artificial neuron and each arrow a connection from the output of one neuron to the input of another.

*Data transformation* methods depend on the specific SL algorithm to be used (Kotsiantis *et al.* 2006). Three common data transformations are scaling, decompositions and aggregations. Many SL methods (e.g. the elastic net regression; Zou & Hastie, 2005) require all predictors to have the same scale such as between 0 and 1. Decomposition may be applied to features that represent a complex concept, as they may be more useful to a ML method when split into their constituent parts (e.g. a date can be split into day, month and year). Aggregation is appropriate when there are features that are more meaningful to the problem when combined into a single feature.

### Training and validation of the model

The data used to run a learning algorithm are called *training* data. In supervised ML the program is told what the output should look like, for example what subjects belong to what category label. A second set of data is called the *test* dataset. Here the labels are again known to the researcher but in this run the program is only given the input data and the task is to correctly assign the outputs or labels. Ideally the test data and the training set should be completely independent but in practice researchers very often randomly split datasets of labelled data in two parts and arbitrarily define one part as the learning data and the other as the test set. If the algorithm is able to estimate correct labels in this new set of cases, i.e. the called *prediction error* is small (e.g. the number of falsely classified cases is much smaller than chance classification), the classifier may be considered to be ‘valid’ to be used in estimating outcomes for cases with unknown outcomes. As elsewhere in classification problems a variety of measures are used to assess prediction accuracy (Steyerberg *et al.* 2010), for example sensitivity (the proportion of correctly classified recovered cases) and specificity (the proportion of correctly not recovered cases) for binary classifications.

Wolpert & Macready (1997) consider that there is unlikely to be a single technique that will always do best for all learning problems. Hand (2006) advocated that we should base our selection on a compromise between the accuracy of the model in predicting outcomes for new cases and the interpretability of the result.

Specific ML terminologies that have been adopted by the SL community are introduced in Table 2. A more detailed set of definitions can be found in (Kohavi, 1998).

**Table 2.** Glossary of statistical/machine learning terms used in this paper

Term	Definition
Feature/attribute/predictor	A numerical (e.g. subset of real values) or categorical (i.e. a finite number of discrete values) value used as input to a learning algorithm
Outcome/response/label	A numerical or categorical value to predict from features
Labelled data	A set of features and labels for an observation
Training set	A collection of data used to train a learning algorithm
Test set	A collection of labelled data
Supervised learning	Techniques used to learn the relationship between independent attributes and a designated dependent attribute (the label)
Unsupervised learning	Learning techniques that group observations without a pre-specified dependent attribute. Clustering algorithms are usually unsupervised
Model	A structure and corresponding interpretation that summarizes or partially summarizes a set of data, for description or prediction. Most learning algorithms generate models that can then be used in a decision-making process
Accuracy	The rate of correct predictions made by the model over a dataset. Accuracy is usually estimated by using an independent test set that was not used at any time during the learning process. More complex accuracy estimation techniques, such as cross-validation and the bootstrap, are commonly used, especially with datasets containing a small number of observations
High-dimensional problem	Problems in which the number of features *p* is much larger than the number of observations *N*, often written *p* > *N*. Such problems have become of increasing importance, especially in genomics and other areas of computational biology
Overfitting	A modelling error that occurs when the model is too closely fit to a limited set of data points. As data being studied often has some degree of error or random noise, an overfitted model is poor in predicting new cases
Multicollinearity	Correlation between features, i.e. the situation where if the value of a feature change, values for the rest of features also change at some degree. When there is multicollinearity between variables in a regression model, its coefficients can become poorly determined and exhibit high variance
*K*-fold cross-validation	A method for estimating the accuracy (or error) of a learning algorithm by dividing the data into *K* mutually exclusive subsets (the ‘folds’) of approximately equal size. *K* models are trained and tested. Each time a model is trained on the data set minus a fold and tested on that fold. The accuracy estimate is the average accuracy for the *K* folds

Table 1 summarizes seven popular SL algorithms. More detailed information about specific learning algorithms can be found elsewhere (Mitchell, 1997, 2006; Vapnik, 1998; Scholkopf *et al.* 2003; Malley *et al.* 2011).

**Fig. 3.**
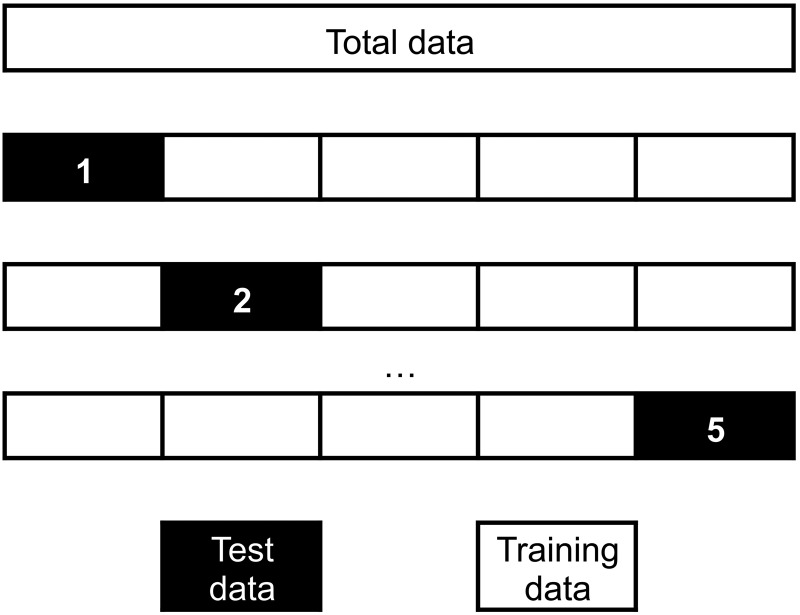
Example of a 5-fold cross-validation. Data are randomly split in 5-fold of equal size. At every step, one fold is selected as test dataset and the remaining four are used as training data. This procedure is repeated five times, selecting in every step a different fold as test data.

A common scheme to train different classifiers and select one based on ability to predict outcomes is the *K*-fold cross-validation (CV). This is a procedure where the original training sample is randomly divided in *K* subsamples, *K*-1 samples are used as a new training set and one is left out as an occasional ‘test’ set in *K* iterations (Fig. 3). The prediction error is then computed across test samples. Minimizing the prediction error from the CV loop is used to select the best algorithm and the best predictive model produced by the same algorithm. CV provides a nearly unbiased prediction error on new observations from the same population (Kohavi, 1995).

### Introducing a generated predictive knowledge to a practical setting

A nice example is provided by the work of Perlis and colleagues (Perlis, 2013) who ran a prospective investigation to identify clinical predictors of antidepressant treatment resistance. The authors selected 15 easy-to-obtain features for patients with known response and adopted a SL approach. Based on the best model obtained, the team developed a web-based clinical decision support system that given the values for the 15 variables for a particular patient suffering from major depression could aid in predicting the risk of being resistant to an antidepressant treatment.

## How statistical learning renders Big Data problems tractable in psychiatric research?

### Dealing with heterogeneous sources of information

Data from different sources (e.g. large longitudinal clinical trials or cohort studies, electronic health records, national health registries) has a greater potential for establishing novel useful ways of categorizing patients’ groups (patient stratification) and for revealing unknown disease correlations compared to learning from each source independently (Shi *et al.* 2012). Specific SL algorithms have demonstrated impressive empirical performance on a wide variety of classification tasks involving heterogeneous Big Datasets (e.g. decision-tree approaches; Breiman, 1984), regularized regression models (Zou & Hastie, 2005), as well as support vector machines (Lewis *et al.* 2006) (Table 1). Integrating such data is a challenge that may include the problem that data are stored in many different formats. However, the handling of Big Data from a variety of sources is becoming ever more feasible and affordable, with many institutions employing their own local clusters of computers (banks of many micro-computers hooked up in parallel and providing huge computational power). ‘Cloud’ computing is another increasingly available option. This refers to using the Internet to access the vast computational resources that are offered commercially by companies such as Amazon, Google and Microsoft.

The IMAGEN study (Whelan *et al.* 2014) is a good example where researchers integrated data from very heterogeneous domains and applied a SL approach of analysis. Domains included brain structure and function, individual personality and cognitive differences, environmental factors, life experiences, and candidate genes. They applied elastic net regularized regression (Zou & Hastie, 2005) to generate models to predict current and future adolescent alcohol misuse based on such holistic characterization. This ‘regularized’ approach automatically dropped out features that were not contributing to the class predictions. Thus the final model incorporated a subset of the most relevant variables for prediction selected from all of the explored families of predictors. The favoured models pointed to life experiences, neurobiological differences and personality as important antecedents of binge drinking, suggesting possible targets for prevention. The authors reported specific predictors in their models along with their regression coefficient as a standard and interpretable measure of strength between each predictor and the outcome. The approach correctly predicted alcohol misuse for individuals not in the original dataset, emphasizing the model's capability to generalize to novel data.

### The search for meaningful predictors of a psychiatric outcome in high-dimensional datasets

Big Datasets in psychiatry research can be ‘Big’ regarding volume and number of features but involving a relative smaller sample size. For example, even though GWAS typically now contain tens of thousands of subjects, there may be many millions of data points. Increasingly large-scale case-control studies also include gene expression, genome sequencing and epigenetics, proteomics or metabolomics inflating the data to research subject ratio even more. This is often called the *high-dimensional data problem*, or the ‘*p* > *N*’ problem (where *p* is the number of features and *N* the number of observations). Such data are commonly represented in a matrix, with more columns than rows. The classical approach of comparing thousands of single association tests and then ranking features by their statistical significance is not an optimal solution. The first concern is that multiple testing increases the risk of spurious findings due to chance. The application of stringent methods to correct this can lead to the detection of strong contributors to outcome at the expense of overlooking smaller contributors. This poses a problem in complex traits and disorders that, by their nature are multifactorial. Another related weakness is that independent analysis variable by variable does not permit inferences about combinations of variables. Generalized linear regression models (Hosmer *et al.* 2013) are problematic in estimating the effect of such combinations. This kind of model is in danger of explaining mainly noise instead of the relationships between variables (and so models are poor in generalizing to new datasets). This problem is known as overfitting (Table 2). A second problem for generalized linear regression is correlation between features, i.e. the situation where if one feature changes, so do one or more other features, an effect known as multicollinearity (Table 2). An example is genetic variation. Due to the fact that most of our genetic information is inherited in ‘blocks’ from our parents, the information at different positions of our genome is expected to be highly correlated within families. Blocks, albeit smaller ones, also occur within genetically homogenous populations. Multicollinearity can seriously distort the interpretation of a model, making it less accurate by introducing bias within the coefficients of the model (Maddala & Lahiri, 2009) and increasing uncertainty, as reflected in inflated standard errors (Glantz & Slinker, 2000; Miles & Shevlin, 2001).

Supervised SL models offer a means to overcome these problems and to maximize the predictive power, hence providing exciting opportunities for individualized risk prediction based on personal profiles (Ashley *et al.* 2010; Manolio, 2013). SL models such as the multivariate adaptive regression splines (MARS) procedure (Friedman, 1991), the CART (Breiman, 1984), elastic net regularized regression (Tibshirani, 1994; Zou & Hastie, 2005; Friedman *et al*. 2010) and support vector machines (Cortes & Vapnik, 1995) (Table 1) perform especially well in the high-dimensional scenario and in the presence of correlation between predictors (Libbrecht & Noble, 2015). They also allow to efficient identification of informative patterns of interactions between clinical and biomarker variables, which are known to play an important role in the development and treatment of many complex diseases (Lehner, 2007; Ashworth *et al.* 2011), but are often missed by single association tests (Cordell, 2009).

### Models in practice: the case of stratified and personalized medicine

In recent years stratified and personalized medicine became of interest in mental health research which utilizes molecular biomarkers (Kapur *et al.* 2012), demographic and clinical information, including patients’ health records, to identify subgroups of patients who are likely to respond similarly to treatment using SL methods. Major depressive disorder is a prime example of a common disorder where there are many available drugs but where there is no straightforward way of deciding which treatment is likely to work for a given individual (Simon & Perlis, 2010). The Genome-based Therapeutic Drugs for Depression (GENDEP Investigators *et al.* 2013) project is a study aiming to test clinical and genetic data as predictors of treatment response to two antidepressant drugs (Uher *et al.* 2009, 2010). The need for prediction at individual level involving hundreds of thousands of variables prompted the use of SL methods (Iniesta *et al.* 2016). The challenge was the integration of clinical with biological markers and deriving optimal models with minimal risk of overfitting. Demographic, clinical and genetic predictors were combined in a model to predict the change in severity symptoms after a 12-week period in a sample of patients randomly treated with one of the two drugs. A linear regularized elastic net model (Zou & Hastie, 2005) looked for the best combination of variables in predicting symptoms course. Interestingly, the feature selection approach of elastic net allowed building drug-specific models that were able to predict treatment outcome with accuracy above a clinical significance threshold. The results suggested a potential for individualized indications for antidepressant drugs. The benefits of using the elastic net were several: first, the elastic net provided an efficient internal method of search and selection of predictors from a large set of variables available. Second, the iterative CV procedure used allowed the selection of predictors based on their ability in predicting outcome for unseen cases, which was the aim of this research. Third, this flexible approach reported distinct and specific models to each outcome and drug sample. Fourth, the elastic net allowed estimation of the combined predictive ability of a high number of variables while preventing the models from overfitting.

The hoped for impact of this type of research is the introduction of a predictive model (last box in Fig. 1) as a clinical decision support system. For a model to be useful in the practical scenario there is a list of challenges we need to overcome. First, the model should have been externally validated in a test dataset. Very often the validation of models built in sample of patients with very specific characteristics (e.g. those coming from randomized clinical trials) is difficult because it is hard to find another similar sample that can work as a ‘test’ dataset. Second, as a consequence, such models tend to poorly generalize to other populations. For example, if a model was built for a homogeneous ethnical population of white Caucasian patients and ethnicity has an effect on outcome, there is no guarantee that such model will predict well for an individual of different ethnicity. Thus some authors argue matching treatments to individuals is a too ambitious aim, as given any model, there can always be a relevant-to-outcome patient characteristic that was not included nor validated. However, it is not all bad news; several studies in cancer were able to find almost perfect biomarkers for treatment selection, specifically for chemotherapy treatment and some progress towards stratified medicine is appearing feasible in psychiatry (Perlis, 2013; Iniesta *et al.* 2016). A third challenge is the generation of easy-to-use tools in the clinical setting. Ideally models should involve a reasonable number of easy-to-obtain variables and be implemented through tools that allow a quick introduction of patients’ data and a simple and clear display of model outputs.

We can conclude that Big Data are becoming a major challenge for statistical analysts in mental health research and a paradigm shift in methods is needed. Statistical learning provides a set of tools that can successfully help in the understanding of such complex datasets. Such methods can be useful as an alternative or in addition to ‘classical’ statistical inference methods based solely on hypothesis testing which has been criticized by many statisticians for many years (Breiman, 2001*a*; Nuzzo, 2014). Big Data analysis and the derivation of predictive SL models for stratified medicine in psychiatry is an emerging and hot area, and such tools have the potential to facilitate a better targeting of interventions and diagnosis of patients.
